# Photodynamic Diagnosis-Guided Ureteroscopic Laser Ablation of Upper Urinary Tract Urothelial Carcinoma: Phase 2, Open-Label, Single-Arm Trial

**DOI:** 10.1245/s10434-024-16351-0

**Published:** 2024-10-14

**Authors:** Takashi Yoshida, Yusuke Motoki, Craig G. Rogers, Johar Raza, Takahiro Nakamoto, Tadashi Matsuda, Hidefumi Kinoshita

**Affiliations:** 1 Department of Urology and Andrology, Kansai Medical University, Osaka, Japan; 2 Graduate School of Engineering, Tottori University, Tottori, Japan; 3 Department of Urology, Osaka Saiseikai-Noe Hospital, Osaka, Japan; 4 Corporate Sponsored Research Programs for Multicellular Interactions in Cancer, Kansai Medical University, Osaka, Japan; 5 Vattikuti Urology Institute, Henry Ford Health, Detroit, MI USA

Recent advancements in devices for ureteroscopy (URS) have enabled endourologists to perform URS with laser ablation of high-volume upper urinary tract urothelial carcinomas (UTUCs) or tumors in challenging locations.^1,2^ However, the high frequency of recurrence at second-look/follow-up URS and the conversion to radical nephroureterectomy (RNU) suggest that the current procedure has limitations such as incomplete ablation.^1–4^ In other words, “successfully resected visually” should be regarded as distinct from “completely resected histologically.” To address this issue, our group previously conducted a pilot study that revealed the dual role of photodynamic diagnosis (PDD) technology—not only as a diagnostic tool, as widely reported,^5^ but also as an intraoperative navigation tool to confirm complete resection.^6^ PDD allows for the identification of tumor cells once they have incorporated 5-aminolevulinic acid hydrochloride (5-ALA), even within coagulated tissues from laser ablation or floating resected tissues, providing a distinct advantage over other enhancement methods such as narrow-band imaging.^6^

In this study, we further evaluated the efficacy and safety of PDD-guided URS using oral 5-ALA.

## Patients and Methods

**Fig. 1:**
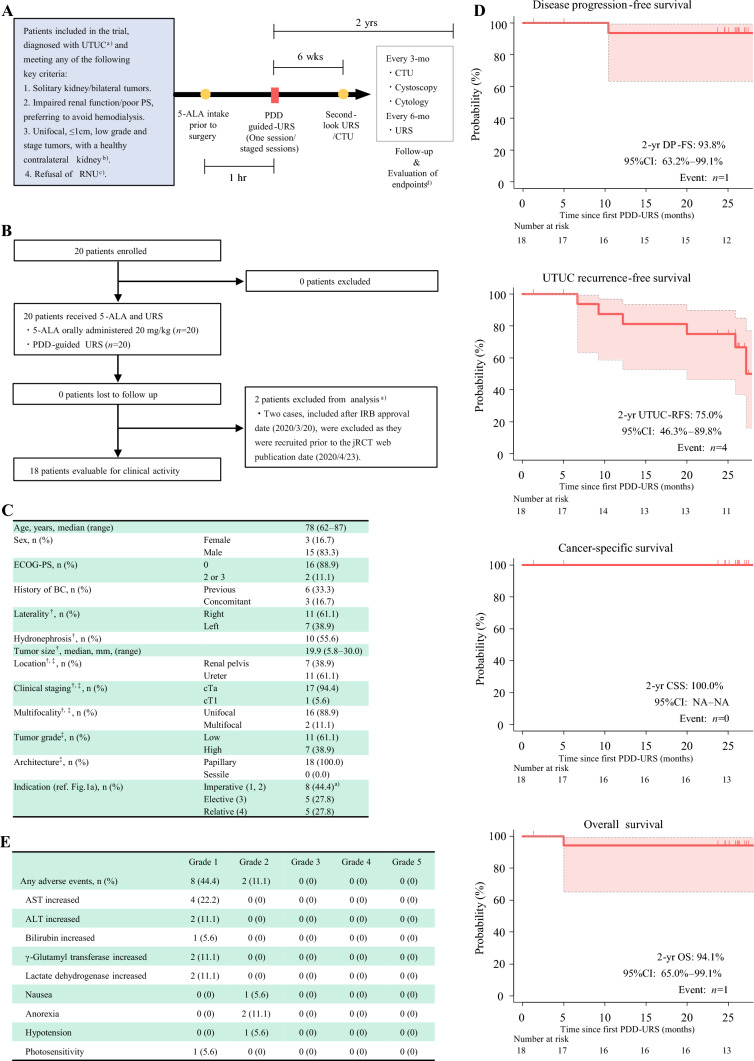
**A** Study outline: ^a^UTUC diagnosis through URS biopsy before inclusion, ^b^elective cases were defined on the basis of a previous version of UTUC guidelines (*Int J Urol*. 2015;22:3-13) upon obtaining IRB approval, ^c^selection of tumors potentially manageable via URS at the surgeon’s discretion, ^d^primary endpoint: disease progression-free survival (DF-PS; defined as the time from the date of first PDD-URS to the date of distant metastasis, the presence of endoscopically unresectable tumors, or upgrading during follow-up for initially low-grade tumors)^1^ and secondary endpoints: ipsilateral UTUC recurrence-free survival (UTUC-RFS; defined as the time from the date of first PDD-URS to the date of ipsilateral UTUC recurrence), cancer-specific survival (CSS; defined as the time from the date of first PDD-URS to the date of death from UTUC), overall survival (OS; defined as the time from the date of first PDD-URS to the date of death from any cause), and safety profile of drug administration; **B** CONSORT diagram, ^a^owing to registration oversight, two cases were excluded from the analysis, three patients evaluated by CTU around 6 weeks post-procedure were unable to undergo second-look URS, with reasons including hip fracture (*n* = 1), coronavirus disease 2019 (COVID-19) infection (*n* = 1), and patient refusal (*n* = 1); **C** baseline patient characteristics assessed via ^†^imaging or ^‡^diagnostic URS. The tumor stage and grade were evaluated according to the 2010 American Joint Committee on Cancer TNM staging system and the 2016 World Health Organization consensus classification, respectively, ^a^solitary kidney (*n* = 4) and bilateral tumors (*n* = 1); **D** Kaplan–Meier curves were analyzed for each endpoint, and Greenwood’s formula was used to calculate 95% CI for survival rates, one patient who experienced disease progression underwent RNU owing to having endoscopically unresectable tumors, and another died because of severe myelodysplastic syndrome, all statistical analyses were conducted using R statistical language version 4.3.1 (R Foundation for Statistical Computing, Vienna, Austria) and EZR version 1.54 (Jichi Medical University Saitama Medical Center, Saitama, Japan); (E) adverse event grades related to 5-ALA administration on the basis of Common Terminology Criteria for Adverse Events version 5.0; *AST* aspartate aminotransferase, *ALT* alanine aminotransferase, *BC* bladder cancer, *ECOG* Eastern Cooperative Oncology Group, *5-ALA* 5-aminolevulinic acid, *UTUC* upper urinary tract urothelial carcinoma, *PS* performance status *PDD* photodynamic diagnosis, *RNU* radical nephroureterectomy, *URS* ureteroscopy, *CI* confidential interval, *CTU* computed tomography urography, *mo* month, *NA* not applicable, *h* hour, *yr* year, *wk* week, *jRCT* Japan Registry of Clinical Trials

After obtaining approval from the National Certified Review Board (jRCTs051200004), we conducted a single-arm, phase 2 trial. The study design and protocol are described in Fig. 1A. Inclusion criteria were patients with age ≥ 20 years and previously confirmed tumor grade via URS biopsy and one of the following: solitary kidney/bilateral tumors; renal dysfunction/poor performance requiring function preservation; unifocal, low-grade and stage tumors with ≤ 1 cm and a healthy contralateral kidney; or refusal of RNU. PDD-guided URS was performed as previously described (https://ars.els-cdn.com/content/image/1-s2.0-S2666168321000720-mmc1.mp4).^6^ Briefly, 5-ALA (20 mg/kg) was dissolved in 50 mL of water and administered orally 60 min before surgery. A semi-rigid or flexible ureteroscope was used to identify and ablate tumors with thulium and holmium: yttrium-aluminum-garnet lasers. Tumor ablation was performed under white light (WL) and PDD, with additional laser ablation if residual tumors showed red fluorescence under PDD. No apparent residual tumors were confirmed under PDD. Hemostasis was performed, and the surgery was completed. The primary endpoint was disease progression-free survival (DP-FS),^1^ and the secondary endpoints included UTUC recurrence-free survival (UTUC-RFS), cancer-specific survival (CSS), overall survival (OS), and the safety profile of drug administration (see legends regarding each definition). In the pilot study cohort, the 2-year PFS rates for the non-PDD-URS group (high-grade cases: 56.2%) and the PDD-URS group (high-grade cases: 40.0%) were approximately 58.0% and 90.0%, respectively.^6^ With a threshold 2-year DP-FS rate of 58.0% and an expected rate of 90.0%, and considering a 2-year registration, 2-year follow-up, two-sided *α*-error of 0.05, 90% power, and a one-sided alternative hypothesis, we determined that at least 14 cases were required. Considering an eventual ineligible rate of 10%, 20 cases were deemed sufficient. If the lower limit of the 95% confidence interval for the 2-year DP-FS rate of PDD-URS exceeded 58.0%, the treatment would be considered significant.

## Results

In total, 20 patients were enrolled from March 2020 to January 2022, and 18 patients completed the study (Fig. 1B). High-grade tumors were observed in seven patients (38.9%) and imperative indications in eight patients (44.4%) (Fig. 1C). Of 16 patients with a single tumor detected by WL diagnostic URS (WL-DxURS), 3 (18.8%) had multiple tumor lesions during PDD evaluation; two (11.1%) patients needed staged sessions owing to multiple large tumors (≥ 15 mm). Only one patient (Clavien–Dindo classification grade II) experienced surgery-related complications. At the second-look URS, conducted for 15 patients, no recurrence was observed. The primary endpoint was met, as the lower limit of the 95% CI for the 2-year DP-FS rate was 63.2%. Moreover, the 2-year DP-FS, UTUC-RFS, CSS, and OS rates were 93.8%, 75.0%, 100.0%, and 94.1%, respectively (Fig. 1D). Although no adverse event of grade ≥ 3 was observed, one patient required a small amount of vasopressor medication for hypotension (Fig. 1C).

## Discussion

The results of this study strengthened the effectiveness of intraoperative PDD navigation by showing improved DP-FS. The identification of lesions missed during WL-DxURS also contributed to complete resection. Furthermore, given the 2-year UTUC-RFS rates of approximately 20–30% reported by experienced institutions,^2,4^ our result of 75% may encourage the utilization of PDD technology to decrease the need for additional laser ablation procedures. Owing to the limited sample size, further studies with multi-institutional cohorts are warranted.

## Data Availability

The data for this clinical trial will be shared on reasonable request to the corresponding author.

## Trial Registration

This trial is registered on Japan Registry of Clinical Trials as jRCTs051200004.
